# Discovering and verifying DNA polymorphisms in a mung bean [*V. radiata *(L.) R. Wilczek] collection by EcoTILLING and sequencing

**DOI:** 10.1186/1756-0500-1-28

**Published:** 2008-06-18

**Authors:** Noelle A Barkley, Ming L Wang, Athey G Gillaspie, Rob E Dean, Gary A Pederson, Tracie M Jenkins

**Affiliations:** 1 USDA-ARS, Plant Genetic Resources Conservation Unit, 1109 Experiment Street, Griffin, GA 30223, USA; 2 University of Georgia, 1109 Experiment Street, Griffin, GA 30223, USA

## Abstract

**Background:**

*Vigna radiata*, which is classified in the family Fabaceae, is an important economic crop and a dietary staple in many developing countries. The species *radiata *can be further subdivided into varieties of which the variety *sublobata *is currently acknowledged as the putative progenitor of *radiata*. EcoTILLING was employed to identify single nucleotide polymorphisms (SNPs) and small insertions/deletions (INDELS) in a collection of *Vigna radiata *accessions.

**Findings:**

A total of 157 DNA polymorphisms in the collection were produced from ten primer sets when using *V. radiata *var. *sublobata *as the reference. The majority of polymorphisms detected were found in putative introns. The banding patterns varied from simple to complex as the number of DNA polymorphisms between two pooled samples increased. Numerous SNPs and INDELS ranging from 4–24 and 1–6, respectively, were detected in all fragments when pooling *V. radiata *var. *sublobata *with *V. radiata *var. *radiata*. On the other hand, when accessions of *V. radiata *var. *radiata *were mixed together and digested with CEL I relatively few SNPs and no INDELS were detected.

**Conclusion:**

EcoTILLING was utilized to identify polymorphisms in a collection of mung bean, which previously showed limited molecular genetic diversity and limited morphological diversity in the flowers and pod descriptors. Overall, EcoTILLING proved to be a powerful genetic analysis tool providing the rapid identification of naturally occurring variation.

## Background

Mung bean (2*n *= 2*x *= 22) [1], also commonly known as green gram or golden gram is classified as *Vigna radiata *(L.) R. Wilczek in the family Fabaceae and placed in the tribe Phaseoleae. Several of the 200 species classified in the genus *Vigna *(including mung bean) are important economic crops that are grown worldwide especially in developing countries in which soil fertility and water are limiting factors. *Vigna *species, in many countries, are used as dietary staples, forage, cover, and green manure crops [2]. The USDA-ARS Plant Genetic Resources Conservation Unit (PGRCU) maintains a germplasm collection that has, as part of its collection, 22 different *Vigna *species. *V. radiata *was chosen as the target species for this study because it is mainly a self-pollinated diploid species with a small nuclear genome size of approximately 520 Mb/1C [3].

The USDA-ARS PGRCU *Vigna radiata *core collection has been evaluated for various morphological characters, but has not been extensively evaluated for molecular genetic variation. The major morphological differences between *V. radiata *var. *sublobata *and *V. radiata *var. *radiata *are the kidney shaped glossy seeds, lobed leaflets, plants prostrate or twining and flowers borne on racemes and opposed in *sublobata *compared to globose seeds, leaflets not lobed, plants erect and not twining and single flowers of *radiata *(J. Wiersema, personal communication). Some of the *V. radiata *var. *radiata *core accessions and one reference plant, *V. radiata *var. *sublobata*, were previously evaluated with SSR markers and morphological descriptors, both of which showed limited genetic diversity (Gillaspie *et al*, unpublished data). Therefore, the purpose of this study was to further assess *V. radiata *accessions for molecular polymorphisms via EcoTILLING and furthermore evaluate the EcoTILLING method for rapid detection of SNPs in plant germplasm.

TILLING (Targeting Induced Local Lesions in Genomes) is a fairly new, innovative, molecular technique that allows rapid identification of induced mutations in a population. This method is a reverse genetics approach that allows scientists to rapidly screen for mutations in a gene of interest without the creation of transgenic material [4,5]. EcoTILLING is a molecular technique that is similar to TILLING, except that it targets natural genetic variation as opposed to induced mutations. This approach allows one to rapidly screen through samples to identify naturally occurring SNPs or small INDELS in a gene of interest. EcoTILLING reduces a scientist's time and effort by weeding out identical haplotypes, and thus, ultimately does not require sequencing all of the individuals in a population to identify polymorphisms. This technique has not been as widely employed as TILLING; however, there are a few published studies on EcoTILLING in plants. This technique was first applied in *Arabidopsis *to uncover 55 haplotypes in five different genes [6]. EcoTILLING was also used to examine DNA variation in populations of black cottonwood (*Populus trichocarpa*) in nine genes and SNPs were detected in all genes examined [7]. Another study utilized EcoTILLING to screen for allelic variation for disease resistance in various *Cucumis species *[8]. Overall, this modified TILLING method has proven to be beneficial to identify natural genetic variation in a gene of interest and to mine for SNPs in plants.

The approach for EcoTILLING in mung bean was to mix DNA from a single reference plant *V. radiata *var. *sublobata *with that of each member of the population (*V. radiata *var. *radiata*) and mine for polymorphic sites (Table 1). The progenitor, *V. radiata *var. *sublobata *was chosen as the reference to ensure that polymorphisms between *radiata *and *sublobat*a would be detected, since intravarietal polymorphisms of *radiata *had been previously difficult to detect with SSR markers. The ten primer sets (Table 2) chosen for this study were designed from *Vigna radiata *sequences deposited in GenBank that were mainly intron spanning targets, which should provide more DNA polymorphisms compared to highly conserved genes where polymorphic sites may be minimal. Intron spanning targets are ideal when assessing polymorphism in a species with high genetic identity.

**Table 1:** Sample list

ID Number	PI Number	Genus	Species	Variety	Core	Collection, Donation or Country of Origin
Reference	220249	*Vigna*	*radiata*	*sublobata*	Yes	Jamaica donated
DS-4	381150	*Vigna*	*radiata*	----	Yes	Thailand donated
DS-6	473636	*Vigna*	*radiata*	----	Yes	Iran developed
DS-18	478618	*Vigna*	*radiata*	----	Yes	Iran developed
DS-22	517909	*Vigna*	*radiata*	----	Yes	Ethiopia donated
DS-23	526222	*Vigna*	*radiata*	----	Yes	Zimbabwe collected
DS-26	165529	*Vigna*	*radiata*	*radiata*	Yes	India collected
DS-31	197019	*Vigna*	*radiata*	*radiata*	Yes	Honduras collected
DS-33	199740	*Vigna*	*radiata*	*radiata*	Yes	Philippines donated
DS-35	201868	*Vigna*	*radiata*	*radiata*	Yes	Iran collected
DS-37	211615	*Vigna*	*radiata*	*radiata*	Yes	Afghanistan collected
DS-40	223280	*Vigna*	*radiata*	*radiata*	Yes	Afghanistan collected
DS-43	239355	*Vigna*	*radiata*	*radiata*	Yes	Zaire donated
DS-50	321115	*Vigna*	*radiata*	*radiata*	Yes	Tanzania collected
DS-51	352723	*Vigna*	*radiata*	*radiata*	Yes	Brazil collected
DS-56	363574	*Vigna*	*radiata*	*radiata*	Yes	India collected
DS-58	363783	*Vigna*	*radiata*	*radiata*	Yes	India collected
DS-61	363945	*Vigna*	*radiata*	*radiata*	Yes	India collected
DS-63	364223	*Vigna*	*radiata*	*radiata*	Yes	India collected
DS-68	377036	*Vigna*	*radiata*	*radiata*	Yes	Iran donated
DS-70	377123	*Vigna*	*radiata*	*radiata*	Yes	Iran donated
DS-80	425002	*Vigna*	*radiata*	*radiata*	Yes	Philippines collected
DS-83	425150	*Vigna*	*radiata*	*radiata*	Yes	Guam collected
DS-84	425178	*Vigna*	*radiata*	*radiata*	Yes	Australia collected
DS-97	425867	*Vigna*	*radiata*	*radiata*	Yes	India collected

Taxonomic classifications of samples used in this study were determined from the NPGS (National Plant Germplasm System) GRIN database (Germplasm Resources Information Network). Accessions in this study are currently classified as either *V. radiata, V. radiata *var. *radiata*, or *V. radiata *var. *sublobata*. Five accessions (PI: 381150, 473636, 478618, 517909, and 526222) included in this study have yet to be classified past the species level.

**Table 2:** List of primers

Name	Target	Forward	Reverse	MgCl_2 _(mM)	AT_m _(°C)
BTF3b	IS	TCAAAAGTCTCCCCGGGGACAAGA	CCAAAGTACAAGCATCTATTGCTGCCA	4.50	61
CDC2	IS	CAACTTTGCAAGGGTGTTGCTTTCT	ACTAACACCTGGCCACACATCTTCA	4.25	65
BP1	unknown	GTTATGGAGTTGATGAGAGGTGTCAGATA	TTGGTAAGTTCTGGAAAATGCCAACCATA	3.75	65
AIGP	IS	CTGATAGGGCCAGGAGGCAGGGAAGA	GTTTTTTAGCATTTGGACGAATGGTTGGT	3.75	60
ATCP	IS	AACCAATTGGTATTGCAGCTCAGAGCCA	TTCCTTGCCAAGAACAAACCGAATGTCA	3.75	65
CALTL	IS	GTGGAAGGCACCATTGATTGACAAC	TCTTCTTCTCAGCCTCTTCAAATGC	3.75	67
MSU380	IS	CACTCATTGCAATTTCCATGCTTCA	CAGTTGTTGTAGCAAGGGCACA	3.75	65
RL3B	unknown	GACACGGTTCTTTGGGATTTCTC	CCTGGCTTTTCGACTTCTCTGAC	3.75	63
DNABP	IS	CAAGACATGGCTCCAATGAG	AAGAGGTAGGCGCTTTTGTG	3.00	65
SHMT	IS	CCAAACAAGGAAAAGAGGTAA	TGACTTATTCACCCCATCCA	4.25	55

A list of the primer sequences including annealing temperature (AT_M_) and final MgCl_2 _concentration (mM) that were used for PCR in this study. The majority of the PCR fragments used are intron spanning (IS); however, some are unknown.

EcoTILLING was performed by amplifying DNA from the fragment of interest in a two fold pooled format, form a heteroduplex from the PCR products by heating (denaturing) and cooling (annealing), applying the endonuclease enzyme CEL I to digest mismatches such as SNPs and INDELS in the heteroduplex, and detect any digested fragments by separation on a LI-COR 4300 DNA analysis system (Additional file 1). Additionally, members of the population were mixed together in a 1:1 ratio to detect intravarietal DNA polymorphisms among *V. radiata *var. *radiata*, since previous work has shown limited genetic diversity. (All intervarietal combinations and ~25% of the total possible intravarietal combinations were carried out in this study). Once variant pools with cleaved fragments were identified, samples were subsequently verified by bidirectional sequencing. Overall, the objectives of this work were to reveal variation among *V. radiata *var. *radiata *accessions and its progenitor *V. radiata *var. *sublobata*, mine for SNPs among *V. radiata *var. *radiata *accessions, and determine the number of different haplotypes in the collection.

### EcoTILLING of *V. radiata *var. *sublobata *with *V. radiata *var. *radiata*

A total of 45 haplotypes ranging from simple to complex were produced from 10 primer sets (Table 2). DNA polymorphisms were observed in every intervarietal pool at each marker examined. The banding pattern of cleaved fragments when digesting heteroduplexes of *V. radiata *var. *sublobata *(reference) and *V. radiata *var. *radiata *(population) tended to be nearly identical when comparing adjacent gel lanes. This suggests that although there are numerous DNA polymorphisms between these two varieties, the *radiata *accessions in this collection are genetically similar and tended to share the exact same sites of variation when pooled with the reference *sublobata*.

**Figure 1:**
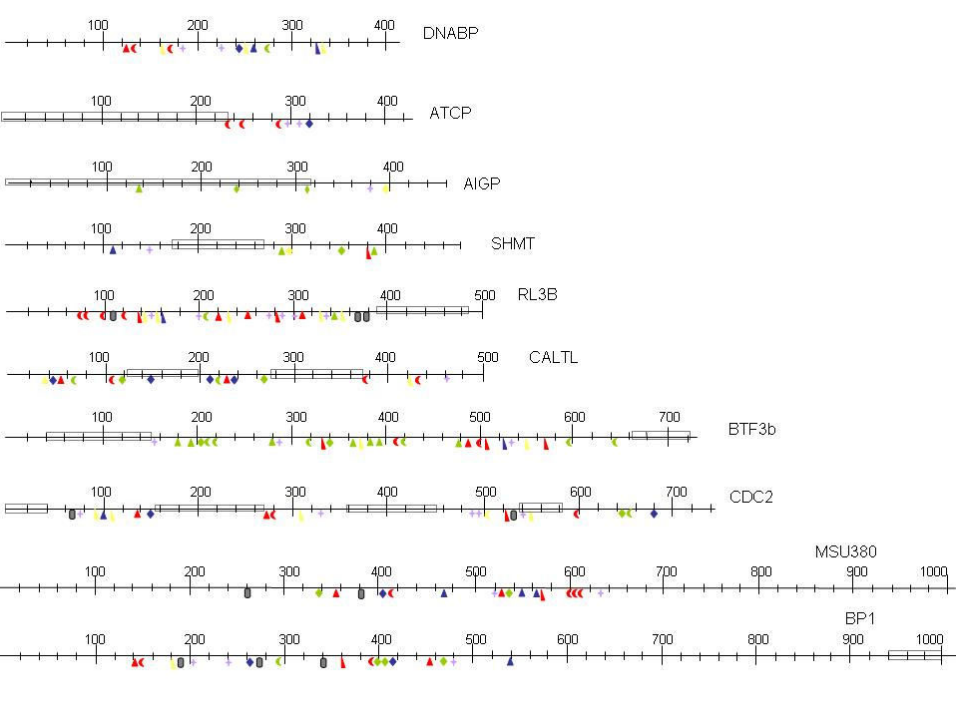
**Map of DNA polymorphisms**. Map of approximate location of the SNPs and INDELS from each of the 10 primer sets examined as determined by EcoTILLING and verified via sequencing. The shape represents the base that was in the reference plant *V. radiata *var. *sublobata *(A = ; C = ; G = ∆; T = ◊). The color represents the base that was observed in one of the members of the population *V. radiata *var. *radiata *(A = green; C = blue; G = yellow; T = red). INDELS are represented by  symbol. Lastly, when comparing the sequence of *V. radiata *var. *sublobata *and *V. radiata *var. *radiata *if two bases in a row were different then a  symbol was placed on the map. The boxes represent areas of the DNA sequence that had high sequence similarity (> 93%) to ESTs, cDNAs or genes deposited in NCBI from *Vigna *species or close relatives such as *Phaseolus *or soybean.

Sequencing positive samples verified the DNA polymorphisms detected by EcoTILLING and revealed a fairly low GC content in all the PCR fragments examined. The mean GC for all 10 primer sets was 36%. Approximately 162 kb of DNA was analyzed via EcoTILLING. The primer sets yielded products ranging from 408 bp to 1109 bp. In general, as the size of the target increased so did the number of DNA polymorphisms detected. The number of DNA polymorphisms ranged from five to 27 in AIGP and BTF3b, respectively (Table 3). The mean number of SNPs and INDELS detected per marker was 13.1 and 2.6, respectively. Overall, 157 DNA polymorphisms were detected when comparing *V. radiata *var. *sublobata *and *V. radiata *var. *radiata *with a mean of 15.7 polymorphisms per marker. The location and type of DNA polymorphisms (SNP/INDEL) observed between *sublobata *and *radiata *are diagramed in Figure 1.

**Table 3:** Marker statistics

Marker	Size (bp)	% GC Content	# of SNPs Intron	# of SNPs Exon	Total # of SNPs	# of INDELS Intron	# of INDELS Exon	Total # of INDELS	Total	% Polymorphic Sites
BTF3b	729	35	24	0	24	3	0	3	27	3.7
CDC2	757	38	16	1	17	4	1	5	22	2.9
BP1	1109	32	16	0	16	3	0	3	19	1.7
AIGP	459	39	1	3	4	1	0	1	5	1.1
ATCP	431	43	4	0	4	2	0	2	6	1.4
CALTL	502	37	14	1	15	1	0	1	16	3.2
MSU380	1087	35	15	0	15	2	0	2	17	1.6
RL3B	501	35	20	0	20	6	0	6	26	5.2
DNABP	408	32	10	0	10	2	0	2	12	2.9
SHMT	478	34	6	0	6	1	0	1	7	1.5
										
Total	6461	--	126	5	131	25	1	26	157	--
Mean	646	36	12.6	0.5	13.1	2.5	0.1	2.6	15.7	2.5

The number of SNP and INDELS detected at each marker between *V. radiata *var. *sublobata *and *V. radiata *var. *radiata*. Total number of SNPs and INDELS are also listed for the putative exon and intron segments determined by high BLAST similarity for each primer set.

The sequence data was used to determine similarity to exons and known genes by BLAST to the non human, non mouse expressed sequence tag (EST) database of NCBI. Most of the examined fragments had regions that had high similarity (>93%) to previously characterized genes, cDNAs, or ESTs from *Vigna *species or close relatives such as *Phaseolus*, suggesting that some of these fragments may contain exons to various genes of interest. The putative exon regions targeted exhibited as expected very few SNPs or INDELS (Table 3 and Figure 1). AIGP had the highest number of SNPs detected in a putative exon region followed by CDC2 and CALTL with 3, 1, and 1, respectively. (The exons of AIGP, CDC2 and CALTL were similar to deposited expressed sequences derived from *Glycine max *auxin-independent growth promoter, *Phaseolus *cyclin dependent protein kinase, and *Phaseolus *calreticulin, respectively). CDC2 was the only marker that had an INDEL detected in an exon. As expected, most of the variation detected came from regions that did not have high similarity to known genes or exons. Further studies will need to be performed to verify if these regions contain exons to genes of interest.

The high level of polymorphism observed between *sublobata *and *radiata *may be due to the fact that the sequence surveyed was mainly non-coding regions and thus, one would expect a higher level of variation due to a lack of selective pressure that is often observed to conserve critical housekeeping genes. Targeting introns also yielded higher levels of DNA polymorphisms (SNPs) and less constraint than conserved exon regions in a study evaluating conserved intron scanning primers (CISP) from eight genera [9]. Another possibility for the high polymorphism level is that the reference DNA *V. radiata *var. *sublobata *could have a high outcrossing rate, which would induce more genetic variation through shuffling of DNA ultimately leading to *V. radiata *var. *sublobata *substantially diverging from *V. radiata *var. *radiata *over evolutionary time. The rate of outcrossing for many of the species in the genus *Vigna *has yet to be determined. Currently, the USDA-ARS *Vigna *germplasm collection only contains one accession of *V. radiata *var. *sublobata*. In a previous study of 115 *V. radiata *var. *sublobata *accessions, a wide range of diversity was identified for agronomic and adaptive traits especially among accessions collected from remote locations [10]. Intravarietal genetic diversity for *sublobata *has also been reported to be high, and furthermore, the Australian form of *sublobata *is considered to be more distantly related to cultivated *radiata *than varieties from Madagascar [11]. Given that only one accession of *V. radiata *var. *sublobata *was available from the USDA germplasm collection with incomplete passport data, it is possible that this accession could be highly divergent from other *V. radiata *var. *sublobata *germplasm and thus, represent more wild type traits. Further work needs to be done to acquire and expand the USDA collection of *V. radiata *var. *sublobata *to look at the possibility and the range of divergence in morphological and genetic traits.

### Variation among *V. radiata *var. *radiata *accessions detected by EcoTILLING

**Figure 2:**
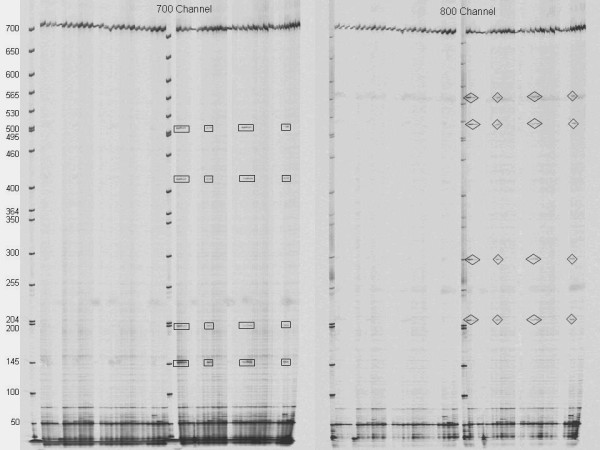
**EcoTILLING image**. LI-COR image of EcoTILLING with *V. radiata *var. *radiata *pooled with *V. radiata *var. *radiata *accessions in a 1:1 ratio and amplified with marker BTF3b along with a 50–700 bp ladder. Six of the ten positive pools that were identified for this primer set are shown in this image with the cleaved fragments marked.

DNA of *V. radiata *var. *radiata *was mixed together in two fold pools to detect intravarietal SNPs and INDELS via EcoTILLING since *V. radiata *var. *radiata *has been shown previously to have limited genetic diversity. Very few SNPs were detected when mixing the population together as compared to pooling *V. radiata *var. *sublobata *(reference) to *V. radiata *var. *radiata*. A total of 52 SNPs were identified and no INDELS were observed among the *V. radiata *var. *radiata *pooled accessions. Marker BTF3b had the most variant pools when comparing *V. radiata *var. *radiata *with 10 positive pools (Figure 2). Four SNPs all at the same position in ten different 2 fold pools were detected in a 729 bp sequence for primer set BTF3b. Markers CDC2 and ATCP also had one and two positive pools with two and three SNPs, respectively. Overall, only 4.1% of the total *V. radiata *var. *radiata *accessions sampled displayed variant pools. However, due to limited time and resources not every possible combination of the population (276 two fold pools for each of the 10 markers) was mixed together so it is possible that some of the SNPs between *V. radiata *var. *radiata *were missed. Future work may include evaluating more intravarietal SNPs for *radiata *accessions. Since polymorphisms were fairly low for most of the primer sets examined, the number of samples in the pool could be increased to enhance throughput and reduce the overall cost.

## Conclusion

Overall, the EcoTILLING method proved to be successful in detecting SNPs and INDELS among *V. radiata *var. *sublobata *(the reference) and members of the population (*V. radiata *var. *radiata*) with numerous polymorphic sites detected at all ten markers examined. However, polymorphisms were less frequent between *V. radiata *var. *radiata *accessions suggesting that these accessions have limited genetic diversity. This lack of polymorphism was also observed in a SSR study (Gillaspie *et al*. unpublished data). It is possible that *V. radiata *var. *radiata *generally has a narrow genetic base or that the collection at USDA-ARS PGRCU needs to be further expanded. Future studies may include developing SNP markers from this data, which can be advantageous for marker assisted selection (MAS) in breeding for specific traits. In general, this method was a powerful tool to uncover SNPs and their approximate location without having to sequence all individuals in the population. This technique is especially useful when working with plants that have a narrow genetic base such as *V. radiata *var. *radiata *or looking for variation in highly conserved genes.

## Competing interests

The authors declare that they have no competing interests.

## Authors' contributions

NAB: EcoTILLING data acquisition, data analysis, and manuscript drafting; MLW: project conception, study design, and manuscript drafting; AGG: sample selection, morphology data, and SSR genotyping; RED: concept design and manuscript drafting; GAP: concept design and manuscript drafting; TMJ: data acquisition, sequence alignments, and manuscript drafting.
